# A screening study on the detection strain of Coxsackievirus A6: the key to evaluating neutralizing antibodies in vaccines

**DOI:** 10.1080/22221751.2024.2322671

**Published:** 2024-02-23

**Authors:** Fan Gao, Pei Liu, Yaqian Huo, Lianlian Bian, Xing Wu, Mingchen Liu, Qian Wang, Qian He, Fangyu Dong, Zejun Wang, Zhongping Xie, Zhongyang Zhang, Meirong Gu, Yingzhi Xu, Yajing Li, Rui Zhu, Tong Cheng, Tao Wang, Qunying Mao, Zhenglun Liang

**Affiliations:** a School of Life Sciences, Tianjin University, Tianjin, People’s Republic of China; b Division of Hepatitis and Enterovirus Vaccines, National Institutes for Food and Drug Control, Beijing, People’s Republic of China; c National Center for AIDS/STD Control and Prevention, Chinese Center for Disease Control and Prevention, Beijing, People’s Republic of China; d Department of Research & Development, Shanghai Institute of Biological Products Co., Ltd, Shanghai, People’s Republic of China; e Department of Research & Development, Taibang Biologic Group, Beijing, People’s Republic of China; f Department of R&D, Wuhan Institute of Biological Products Co., LTD, Wuhan, People’s Republic of China; g Department of Production Management, Institute of Medical Biology, Chinese Academy of Medical Sciences, Kunming, People’s Republic of China; h The Second Research Laboratory, National Vaccine and Serum Institute, Beijing, People’s Republic of China; i R&D Center, Minhai Biotechnology Co., LTD, Beijing, People’s Republic of China; j R&D Center, Sinovac Biotech Co., LTD, Beijing, People’s Republic of China; k State Key Laboratory of Molecular Vaccinology and Molecular Diagnostics, School of Public Health, Xiamen University, Xiamen, People’s Republic of China

**Keywords:** Coxsackievirus A6, hand foot and mouth disease, vaccine, cross-neutralization, cross-protection

## Abstract

The increasing incidence of diseases caused by Coxsackievirus A6 (CV-A6) and the presence of various mutants in the population present significant public health challenges. Given the concurrent development of multiple vaccines in China, it is challenging to objectively and accurately evaluate the level of neutralizing antibody response to different vaccines. The choice of the detection strain is a crucial factor that influences the detection of neutralizing antibodies. In this study, the National Institutes for Food and Drug Control collected a prototype strain (Gdula), one subgenotype D1, as well as 13 CV-A6 candidate vaccine strains and candidate detection strains (subgenotype D3) from various institutions and manufacturers involved in research and development. We evaluated cross-neutralization activity using plasma from naturally infected adults (*n* = 30) and serum from rats immunized with the aforementioned CV-A6 strains. Although there were differences between the geometric mean titer (GMT) ranges of human plasma and murine sera, the overall trends were similar. A significant effect of each strain on the neutralizing antibody test (MAX/MIN 48.0 ∼16410.3) was observed. Among all strains, neutralization of the S112 strain by 15 different sera resulted in higher neutralizing antibody titers (GMT_S112 _= 132.0) and more consistent responses across different genotypic immune sera (MAX/MIN = 48.0). Therefore, S112 may serve as a detection strain for NtAb testing in various vaccines, minimizing bias and making it suitable for evaluating the immunogenicity of the CV-A6 vaccine.

## Introduction

**Figure 1.**
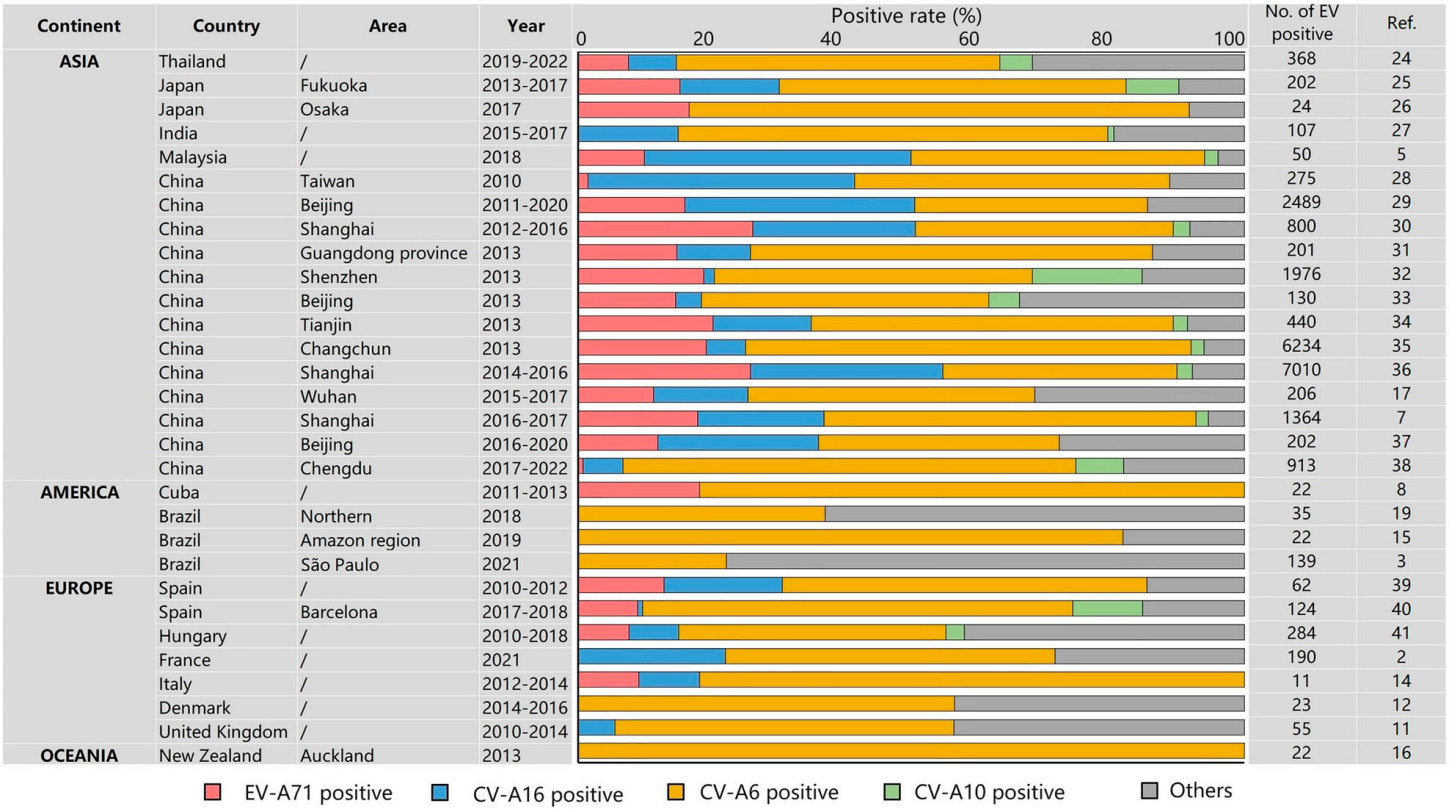
Epidemiology of HFMD since 2010.

Coxsackievirus A6 (CV-A6) is a small icosahedral RNA virus belonging to the human Enterovirus A species (EV-A) within the Picornaviridae family [1]. It is associated with a spectrum of human diseases, ranging from mild conditions, such as hand, foot, and mouth disease (HFMD) [2–8] and herpangina [9,10], to more severe manifestations, such as atypical HFMD [11–15], onychomadesis [12,15,16], and rare neurological complications [6,16–19]. These conditions can affect individuals of all ages, including children and adults [11,12,16,20,21]. Historically, HFMD outbreaks have predominantly been caused by Enterovirus 71 (EV-A71) and CV-A16 [22,23]. After 2010, CV-A6 has become the leading viral pathogen in numerous countries, causing HFMD outbreaks [2–41] (Figure 1). Han et al. [42] analyzed HFMD surveillance data in Jiangsu Province, China, from 2009 to 2020 and identified the emergence of CV-A6 as a pathogen causing severe cases in 2017. This proportion gradually increased, and by 2020, it accounted for approximately one-third of all pathogens causing severe HFMD, resulting in a large burden on individuals. Vaccines are a highly efficacious approach for mitigating disease severity and reducing mortality.

Since December 2015, HFMD vaccines, including inactivated whole- virus EV-A71 vaccines manufactured by three Chinese manufacturers, have been approved for marketing to control severe and lethal HFMD [43,44]. However, developing of a monovalent EV-A71 vaccine is only an initial step toward comprehensive HFMD control [44]. Owing to the limited cross-protection between different EV-A serotypes [44], relying solely on a monovalent EV71 vaccine cannot adequately control CV-A6-induced HFMD. Currently, global institutions are endeavoring to develop CV-A6 vaccines [45–50]. Zhou et al. [45] produced a monovalent CV-A6 virus-like particle (VLP) vaccine using *Pichia pastoris* yeast transformed with a vector encoding both the P1 and 3CD proteins of CV-A6. Immunization with CV-A6 VLPs elicited CV-A6-specific serum antibodies in mice. Passive transfer of anti-VLP sera protected recipient mice against lethal CA6 challenge. Additionally, studies have been conducted to develop multivalent HFMD vaccines containing CV-A6 by mixing different EV monovalent vaccines, including bivalent [46], trivalent [47–49], and quadrivalent vaccines [50] (Table 1).

**Table 1.** Summary of the research and development of the CV-A6 vaccine

Vaccine	Year	Component	Type	CV-A6 strain	CV-A6	Cell	Stage	Institution	Country	Ref.
					GenBank ID				/Region	
Monovalent	2016	CV-A6	VLP	SZc173/13	KF682362.1	*Pichia pastoris*	Pre-clinical	Institut Pasteur of Shanghai Chinese Academy of Sciences	Mainland China	[45]
Bivalent	2018	CV-A6, CV-A10	INA	/	/	RD	Pre-clinical	Taishan Medical University	Mainland China	[46]
Trivalent	2015	EV-A71, CV-A16, CA6	INA	Gdula	AY421764.1	RD/Vero	Pre-clinical	University of Wisconsin-Madison	United States	[47]
	2016	EV-A71, CV-A16, CA6	INA	M0746	/	RD	Pre-clinical	National Health Research Institutes	Taiwan, China	[48]
	2018	CV-A6, CV-A10, CV-A16	INA	/	/	RD/Vero	Pre-clinical	National Institute of Health	South Korea	[49]
Quadrivalent	2018	EV-A71, CV-A16, CV-A6, CV-A10	VLP	SZc173/13	KF682362.1	Sf9	Pre-clinical	Institut Pasteur of Shanghai Chinese Academy of Sciences	Mainland China	[50]
	/	EV-A71, CV-A16, CV-A6, CV-A10	INA	/	/	/	Pre-clinical	Sinovac Biotech Co., Ltd.	Mainland China	/
	/	EV-A71, CV-A16, CV-A6, CV-A10	INA	/	/	/	Pre-clinical	Wuhan Institute of Biological Products Co., Ltd.	Mainland China	/
	/	EV-A71, CV-A16, CV-A6, CV-A10	INA	/	/	/	Pre-clinical	Minhai Biotechnology Co., Ltd.	Mainland China	/
	/	EV-A71, CV-A16, CV-A6, CV-A10	INA	/	/	/	Pre-clinical	Wantai BioPharm/Xiamen University	Mainland China	/
	/	EV-A71, CV-A16, CV-A6, CV-A10	INA	/	/	/	Pre-clinical	National Vaccine and Serum Institute	Mainland China	/
	/	EV-A71, CV-A16, CV-A6, CV-A10	VLP	/	/	/	Pre-clinical	Huasong (Shanghai) Biomedical Technology Co., Ltd.	Mainland China	/

INA: Inactivated vaccine. VLP: Virus like particles vaccine. /: Not available.

The correlation between neutralizing antibodies (NtAbs) and protection has been consistently demonstrated in numerous studies on enteroviruses [43,44,51–55], emphasizing the pivotal role of accurate detection of neutralizing antibodies in vaccine evaluation. Therefore, objective and accurate evaluation of the NtAb response level to different vaccines is challenging, given the concurrent development of multiple vaccines in China and worldwide [45–50], each utilizing distinct vaccine strains. The choice of the detection strain is a crucial factor that influences the detection of NtAbs. Research on EV-A71 [51] and CV-A10 [56] has demonstrated that differences in test results can be as high as 384-fold and 4096-fold, respectively, when using distinct detection strains. However, this topic has not yet received sufficient attention. In this study, the National Institutes for Food and Drug Control (NIFDC), a national laboratory for vaccine quality control in China, collected a prototype strain (Gdula), one subgenotype D1, as well as 13 CV-A6 candidate vaccine strains and candidate detection strains (subgenotype D3) from various institutions and manufacturers involved in research and development (R&D). We identified a detection strain that exhibited higher NtAb titers and more consistent responses across different genotypic immune serum samples. It has the potential to serve as a detection strain for NtAb testing in various vaccines, minimizing bias and making it suitable for evaluating the immunogenicity of the CV-A6 vaccine.

Moreover, an effective vaccine should be able to induce a broad spectrum of neutralizing activities against various strains [51]. However, obtaining broad-spectrum efficacy data is challenging because single manufacturers have a limited number of strains. This study aimed to compare the cross-neutralization activities of antibodies induced by 15 CV-A6 strains in order to understand the differences among various viral strains and provide valuable reference data for manufacturers regarding the broad-spectrum efficacy of these strains.

## Materials and methods

### Viruses and cells

A total of 15 CV-A6 viruses were collected. Gdula was purchased from the American Type Culture Collection (ATCC). XM and S101 were propagated from viral samples preserved at the NIFDC. Others were kindly provided by the Institute of Medical Biology, Chinese Academy of Medical Sciences, National Vaccine and Serum Institute, Wuhan Institute of Biological Products Co., Ltd., Minhai Biotechnology Co., Ltd., and Sinovac Biotech Co., Ltd.. All viruses were propagated in rhabdomyosarcoma (RD) cells using MEM solution (Gibco, USA) containing 2% (v/v) fetal bovine serum (Gibco), 2 mM L-glutamine (Gibco), and 100 IU/ml penicillin and streptomycin (Gibco). The 50% cell culture infective dose (CCID_50_) was determined using RD cells, and the values were calculated using the Karber formula [57]. Details of the viral strains used in this study are presented in Table 2.

The CV-A6 sequences in the VP1 region were downloaded from the National Center for Biotechnology Information (NCBI). Sequences containing clear information, such as isolation time and location, were selected for further analysis, and the VP1 region sequences were subjected to cluster analysis using a clustering similarity threshold of 0.97. During the same isolation year, 1–5 sequences were selected as representative sequences from the same sampling country, region, or province, and 364 CV-A6 VP1 sequences containing accurate sampling information were obtained. A phylogenetic tree was constructed using the VP1 sequences of 15 CV-A6 candidate vaccine strains and the candidate detection strains were collected, and 364 viral sequences were curated. MEGA7 software was used for genotyping. A distance of less than 0.15 between groups was considered the same genotype, and a distance of less than 0.08 was considered the same subgenotype. Representative sequences of each genotype and 15 CV-A6 sequences were selected, and the Maximum Likelihood (ML) method was used to construct a phylogenetic tree. First, the optimal nucleic acid replacement model was calculated as SYM + R4 using IqTree 1.6.2. An ML tree was constructed using a bootstrap value of 1000. We tagged the ML tree using Adobe Illustrator CC 2019 23.0. Homology was analyzed using an online tool (https://www.ebi.ac.uk/Tools/msa/Clustalo/).

### Animal

Wistar rats and one-day-old suckling mice (BALB/c) were purchased from the Laboratory Animal Resources Institute, NIFDC. All animal experiments were approved by the NIFDC Ethics Committee before the commencement of the study [NIFDC-2020(B)018] and [NIFDC-2023(B)044].

### Serum samples

#### Plasma

Thirty plasma samples collected from naturally infected adults between January and May 2019 in Cao County, Shandong Province, China, were gifted by the Taibang Biologic Group. Written informed consent was obtained from the guardians of all donors.

#### Serum samples from rats inoculated with different CV-A6 viruses

Anti-Gdula, anti-XM, anti-S101, anti-S102, anti-S104 to S110, anti-S112, and anti-S114 sera were collected from female Wistar rats (specific pathogen free) aged 6–8 weeks and immunized with the respective CV-A6 strains (three rats/strain) via the intraperitoneal (i.p.) route at an approximate dose of 10^7.5^ CCID_50_. Immunizations were performed at weeks 0, 2, and 4, and blood samples were collected at week 5 and centrifuged at 4,000 × *g* at 2–8 °C for 30 min to obtain sera. The sera were stored at −20 °C. Anti-S113 and anti-S103 serum samples were provided by the Wuhan Institute of Biological Products Co., Ltd.

### Measurement of CV-A6 cross-NtAbs

CV-A6 NtAb levels in the serum samples (or plasma samples) were measured using the cytopathogenic effect (CPE) method. Two-fold serial dilutions of serum (started at l:8) were incubated with an equal volume of virus culture (50 μL) containing 100 CCID_50_/well of CV-A6 strains at 37 °C for 2 h. RD cell suspension (final concentration: 2.0 × 10^5^ cells/mL) was then added, and the mixture was incubated in a CO_2_ incubator at 35 °C for seven days before the CPE was observed using microscopy. Neutralizing titers were defined as the highest dilutions required to achieve 50% CPE inhibition.

### Cross-protection

BALB/c suckling mice aged 1 d (5–8/group) were challenged by lethal doses (30 LD_50_) of 20 μL via i.p., followed by injection with the diluted protective serum via i.p. within 1 h. After 21 days of continuous observation, deaths were recorded to evaluate the passive protective effects of the serum *in vivo*.

### Statistical analysis

Microsoft Excel 2020, SPSS Statistics 21, and GraphPad Prism 8.0 were used for statistical processing and data analyses. A CV-A6 NtAb titer <8 was considered negative, whereas a CV-A6 NtAb titer ≥8 was considered positive. The NtAb titer <8 was set to 4 during the calculation. NtAb titers were log-transformed to calculate the geometric mean titer (GMT). The value of MAX/MIN refers to the ratio of the maximum and minimum values of NtAbs against each strain detected in the serum or plasma. The NtAb of each antiserum against counterpart strain was unified by multiplying a coefficient and shown as 100 eventually, and NtAbs against other strains were calculated using the same calculation. The LD_50_ was calculated using the Karber formula [57].

## Results

### Genotyping

**Figure 2.**
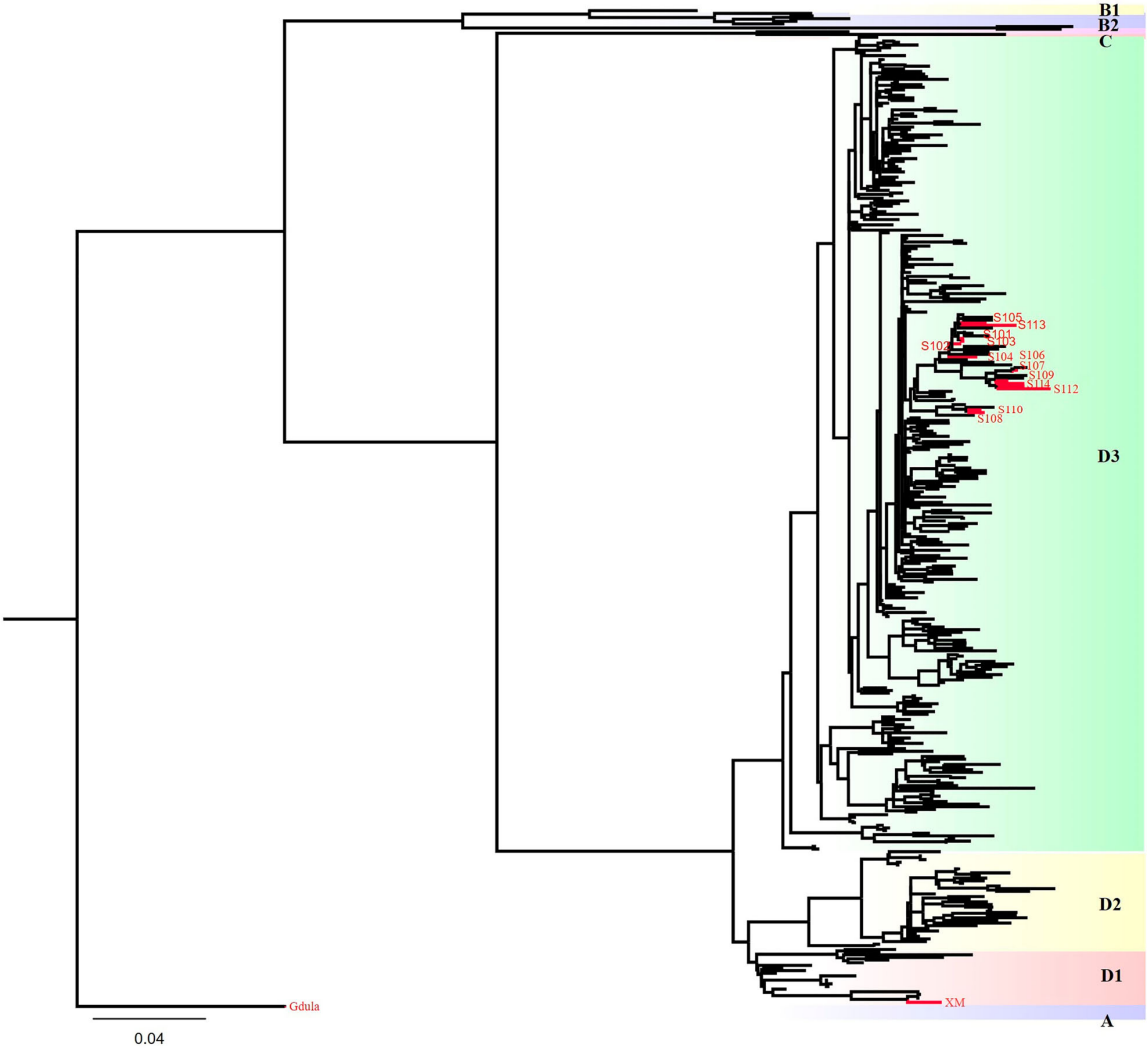
The genotyping of the 15 CV-A6 vaccine and detection candidate strains used in this study. The ML tree analysis revealed that the prototype Gdula strain belongs to genotype A, while the 13 CV-A6 strains belong to subgenotype D3, and the XM strain belongs to subgenotype D1. The names of these strains are indicated in the figure.

The prototype A/Gdula was purchased from ATCC, and 14 CV-A6 strains were obtained from institutions and manufacturers involved in R&D in China. These strains were isolated from different regions of China between 2016 and 2019. In total, 364 CV-A6 VP1 sequences were downloaded from the NCBI database. A phylogenetic tree was constructed using the VP1 sequences of the 15 CV-A6 strains collected in this study and 364 viral strains obtained from the NCBI database (Figure 2). The results showed that 13 strains isolated from mainland China from 2016 to 2019 belonged to the dominant D3 subgenotype branch, and the XM strain isolated from Chinese Taiwan belonged to the dominant D1 subgenotype branch. In addition, the D3 subgenotype exhibited a range of 94.21%–99.78% sequence identity among its members while showing the identity of 82.19%–83.61% with genotype A and 88.52%–90.05% with the D1 subgenotype. The homology between A and D1 was 82.40%.

### Screening detection strain

#### Cross-neutralization activities of different CV-A6 strains on the plasma of naturally infected humans

**Figure 3.**
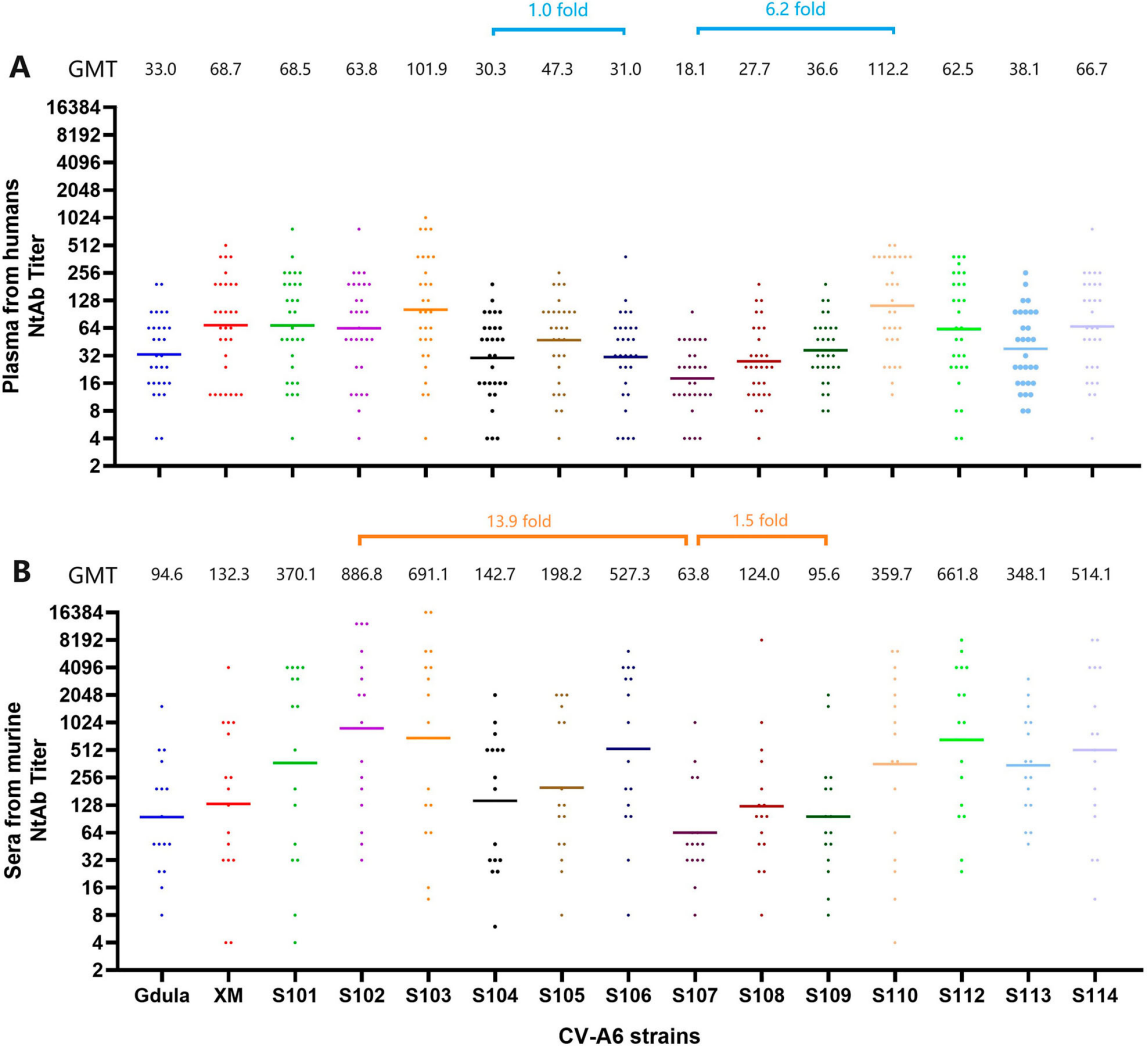
Cross-neutralizing capacity of the plasma from humans naturally infected with CV-A6 and the sera from murine immunized with CV-A6. A: The GMTs of 30 human plasma against various strains of CV-A6 ranged from 18.1 to112.2. The difference among the GMTs for genotype A, subgenotype D1, and C3 strains ranged from 1.0–6.2-folds (shown with a blue bar). B: The GMTs of 15 murine sera against various strains ranged from 63.8–886.8, of which the discrepancy among all strains was 1.5–13.9-fold (shown with an orange bar).

Cross-neutralizing antibody activity against CV-A6 strains was assessed in 30 plasma samples collected from naturally infected humans living in Cao County, Shandong Province, mainland China, between January and May 2019. Fifteen CV-A6 strains were used to detect the NtAbs. As shown in Figure 3(A), the neutralization activity of plasma against all strains varied, with GMT of neutralizing antibodies against all strains ranging from 18.1–112.2. The variation ranged from 1.0- to 6.2-fold (GMT_S106_/GMT_S104 _= 1.0, GMT_S110_/GMT_S107 _= 6.2); the variation between subgenotype D3 and subgenotype D1 ranged from 0.3- to 1.6-fold (GMT_S107_/GMT_XM_ = 0.3, GMT_S110_/GMT_XM_ = 1.6); the variation between subgenotype D3 and genotype A ranged from 0.5- to 3.4-fold (GMT_S107_/GMT_Gdula_ = 0.5, GMT_S110_/GMT_Gdula_ = 3.4); and the variation between subgenotype D1 and genotype A was 2.1-fold (GMT_XM_/GMT_Gdula_). The order of GMTs against the different strains, from low to high, was as follows: S107 < S108 < S104 < S106 < Gdula < S109 < S113 < S105 < S112 < S102 < S114 < S101< XM < S103 < S110. The highest NtAb GMT was observed for S110 (GMT_S110_ = 112.2), and the lowest GMT was observed for S107 (GMT_S107_ = 18.1), indicating that S107 was more difficult to be neutralized than the other strains. Among the remaining 14 strains, there was a maximum fold variation of 3.7 (GMT_S110_/GMT_S104_), suggesting a certain degree of similarity between these strains.

#### Cross-neutralization activities of different CV-A6 strains in murine sera

Serum samples were collected after immunizing Wistar rats with the 15 CV-A6 strains separately. Each serum sample was tested to determine its cross-neutralization ability against various CV-A6 strains. Although there were differences between the GMT ranges of the human plasma and murine sera, the overall trends were similar. As shown in Figure 3(B), the GMTs of 15 sera detected with 15 strains ranged from 63.8–886.8, with a 1.5- to 13.9-fold variation (GMT_S109_/GMT_S107_ = 1.5, GMT_S102_/GMT_S107_ = 13.9). The variation between subgenotypes D3 and D1 ranged from 0.5- to 6.7-fold (GMT_S107_/GMT_XM_ = 0.5, GMT_S102_/GMT_XM_ = 6.7); the variation between subgenotype D3 and genotype A ranged from 0.7–9.4 (GMT_S107_/GMT_Gdula_ = 0.7, GMT_S102_/GMT_Gdula _= 9.4); and the variation between subgenotype D1 and genotype A was 1.4-fold (GMT_XM_/GMT_Gdula_). The order of GMTs against the different strains, from low to high, was as follows: S107 <Gdula < S109 < S108 < XM < S104 < S105 <S113 < S110 < S101 < S114 < S106 < S112 <S103 < S102. Among the D3 strains, S107 showed the lowest propensity to be neutralized compared with the other strains of subgenotype D3. Further investigation is warranted to elucidate the underlying mechanisms.

To mitigate the error originating from the different titers of each serum, the NtAb titers of each antiserum against its counterpart strain were standardized by converting them proportionately to 100 while appraising the impact of each strain on NtAb detection. As shown in Table 3, GMTs of the detection strains ranged from 11.7–176.9, and the fold was 15.1 over the GMT of S107, which had the lowest value (GMT_S107_ = 11.7) among all strains tested followed by S109 (GMT_S109_ = 17.6) and Gdula (GMT_Gdula_ = 17.8), indicating that genotype A and subgenotype D3/S109 were also hard to be neutralized. Conversely, S102, S112, and S106 exhibited the highest GMT values (176.9, 132.0, and 111.4, respectively), indicating their susceptibility to neutralization. The MAX/MIN values of each strain against all sera were calculated to screen for homogeneously reactive strains with any serum (Table 3). Screening of the detection strain was conducted to evaluate its susceptibility to neutralization by different sera, focusing on minimizing the MAX/MIN value, which indicated equal and desirable neutralization potential. The results demonstrated that the MAX/MIN of S103 was the highest (16410.3), indicating that the relative discrepancies in the neutralization of S103 by different sera were the most evident. Conversely, that of strain S112 was minimal (48.0). This suggests that S112 exhibits a more consistent response across all sera and may serve as a detection strain for different vaccines.

**Table 2.** List of CV-A6 strains.

Name	Genotype	Year	Location^a^	Virus titer (lgCCID_50_/mL)	LD_50_ (lgCCID_50_/mL)
Gdula	A	1949	USA	7.62	3.62
XM	D1	2007	Taiwan province	8.17	N/A
S101	D3	2016	Hubei province	8.31	1.78
S102	D3	2016	Guangdong province	9.38	3.21
S103	D3	2016	Hubei province	8.44	N/A
S104	D3	2017	Beijing	8.38	N/A
S105	D3	2017	Beijing	8.44	N/A
S106	D3	2018	Beijing	8.63	N/A
S107	D3	2018	Beijing	8.31	N/A
S108	D3	2018	Beijing	8.31	N/A
S109	D3	2018	Beijing	9.44	N/A
S110	D3	2018	Guangdong province	8.94	2.19
S112	D3	2018	Hubei province	8.88	4.23
S113	D3	2018	Hubei province	7.85	N/A
S114	D3	2019	Guangdong province	8.50	N/A

^a^ Gdula was isolated from the USA, whereas the others were isolated from China. LD_50_: median lethal dose. N/A: not applicable.

**Table 3.** Comparison of serum cross-neutralization capacities after setting the value of serum neutralization against the corresponding virus strain to 100.

Murine Sera	CV-A6 stains														
	Gdula	S101	XM	S106	S107	S110	S112	S114	S102	S104	S105	S108	S109	S113	S103
anti-Gdula	**100.0**	33.3	33.3	200.0	33.3	25.0	400.0	100.0	266.7	33.3	50.0	50.0	33.3	133.3	12.5
anti-S101	9.4	**100**.**0**	18.8	25.0	1.2	100.0	100.0	100.0	50.0	12.5	50.0	3.1	4.7	25.0	1.6
anti-XM	75.0	75.0	**100**.**0**	150.0	75.0	300.0	150.0	300.0	200.0	50.0	75.0	75.0	150.0	200.0	25600.0
anti-S106	50.0	12.5	12.5	**100**.**0**	50.0	12.5	100.0	37.5	200.0	18.8	25.0	25.0	25.0	200.0	50.0
anti-S107	75.0	100.0	100.0	400.0	**100**.**0**	100.0	400.0	100.0	600.0	75.0	100.0	75.0	75.0	200.0	9600.0
anti-S110	25.0	50.0	66.7	33.3	16.7	**100**.**0**	33.3	133.3	66.7	16.7	33.3	133.3	33.3	50.0	266.7
anti-S112	4.7	75.0	6.3	75.0	1.2	50.0	**100**.**0**	18.8	150.0	6.3	3.1	2.3	1.6	18.8	4.7
anti-S114	6.3	50.0	12.5	50.0	3.1	75.0	100.0	**100**.**0**	150.0	25.0	18.8	12.5	18.8	12.5	75.0
anti-S102	1.6	12.5	2.1	50.0	0.4	6.3	33.3	12.5	**100**.**0**	1.6	1.6	0.8	0.8	3.1	33.3
anti-S104	6.3	16.7	25.0	25.0	8.3	50.0	33.3	66.7	50.0	**100**.**0**	16.7	25.0	25.0	33.3	266.7
anti-S105	1.2	200.0	6.3	200.0	3.1	50.0	300.0	200.0	600.0	25.0	**100**.**0**	18.8	12.5	100.0	3.1
anti-S108	400.0	3200.0	800.0	3200.0	200.0	2400.0	800.0	3200.0	2400.0	400.0	800.0	**100**.**0**	37.5	150.0	4800.0
anti-S109	75.0	800.0	50.0	600.0	50.0	600.0	1600.0	600.0	1600.0	75.0	150.0	150.0	**100**.**0**	600.0	200.0
anti-S113	18.8	75.0	18.8	37.5	12.5	25.0	37.5	50.0	18.8	12.5	37.5	25.0	18.8	**100**.**0**	50.0
anti-S103	4.7	37.5	25.0	75.0	9.4	37.5	50.0	18.8	50.0	12.5	25.0	12.5	6.3	37.5	**100**.**0**
GMT	17.8	76.4	26.7	111.4	11.7	72.1	132.0	98.9	176.9	25.7	36.5	22.1	17.6	63.5	107.6
MAX/MIN	341.9	256.0	384.6	128.0	512.8	384.0	48.0	256.0	128.0	256.4	512.8	192.3	192.3	191.7	16410.3

### The difference in broad-spectrum efficacy among CV-A6 strains

**Figure 4.**
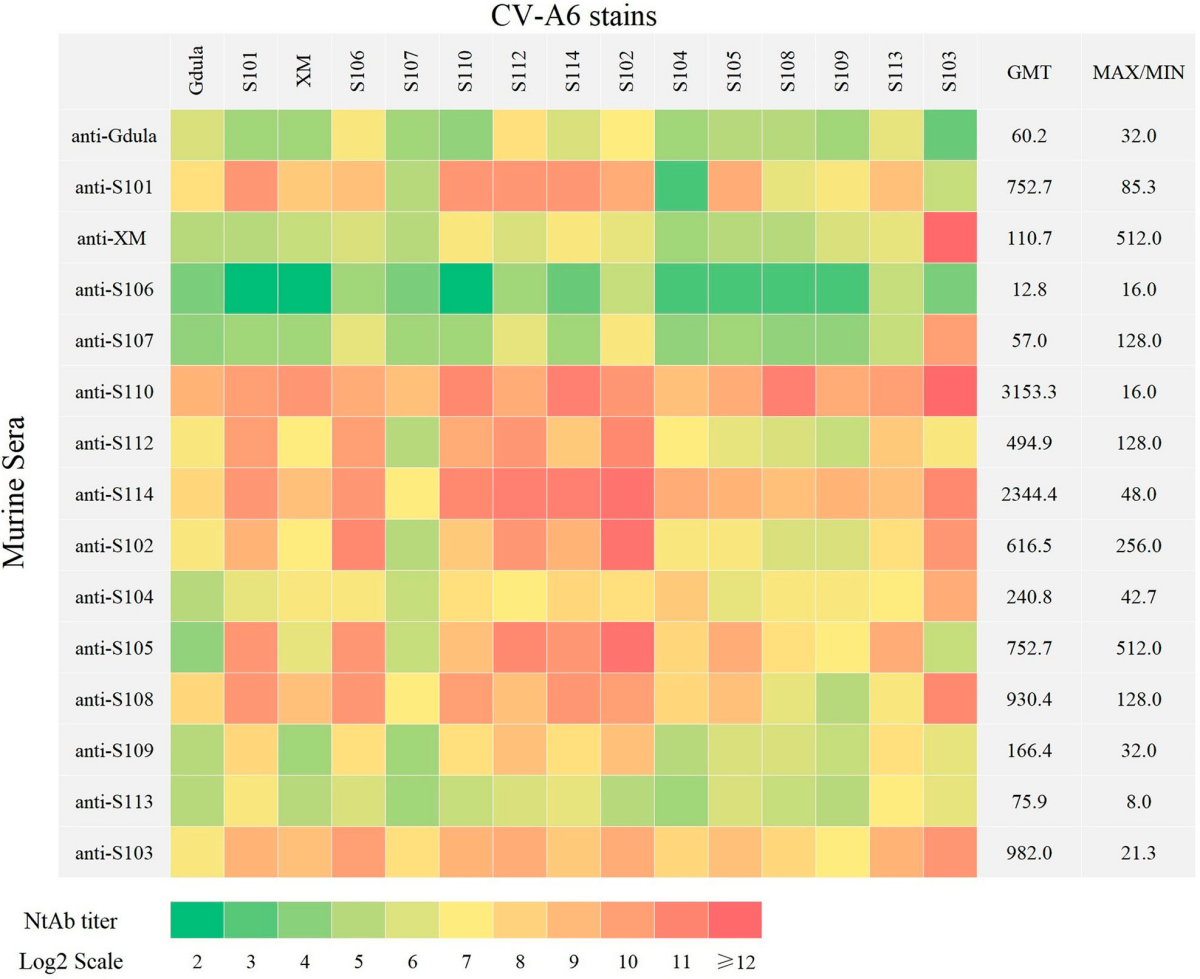
Cross-neutralization ability of sera from murine inoculated with CV-A6 strains. Fifteen sera were collected after inoculating murine with 15 CV-A6 strains separately. CV-A6 NtAb titer < 8 (Log2 Scale < 3) was considered negative, while CV-A6 NtAb titer ≥ 8 (Log2 Scale ≥ 3) was considered positive.

As shown in Figure 3, the NtAb titer of the serum immunized by Gdula (anti-Gdula) and XM (anti-XM) was positive for all 15 CV-A6 strains (NtAb titer ≥ 8), suggesting that the immune sera of A and D1 had cross-neutralized with all subgenotype D3 strains. The broad-spectrum neutralization activity of anti-S106 was found to be limited as it could not neutralize three out of the 15 CV-A6 strains tested (NtAb titer < 8); the NtAb titer of anti-S110 against all strains was positive, and the GMT was the highest (3153.3). Cross-neutralizing homogeneity was assessed by calculating the MAX/MIN value of each serum sample for all strains (Figure 4). A smaller MAX/MIN value indicated better cross-neutralizing ability of the serum derived from mice immunized with this strain. The MAX/MIN values of all the strains ranged from 8.0–512.0, indicating differences in the cross-neutralizing homogeneity of each serum. The sequence of MAX/MIN values from lowest to highest was as follows: anti-S113 < anti-S110 = anti-S106 < anti-S103 < anti-Gdula = anti-S109 < anti-S104 < anti-S114 < anti-S101 < anti-S112 = anti-S107 = anti-S108 < anti-S111 = anti-S102 < anti-XM = anti-S105. The MAX/MIN values of anti-XM and anti-S105 were the highest (512.0), suggesting that the cross-neutralization ability of these two sera varied against different strains. The MAX/MIN values of anti-S113, anti-S110, and anti-S106 were the lowest (8.0, 16.0, and 16.0, respectively), indicating that S113, S110, and S106 may have high neutralizing activity, broad-spectrum activity, and cross-neutralizing homogeneity.

**Figure 5.**
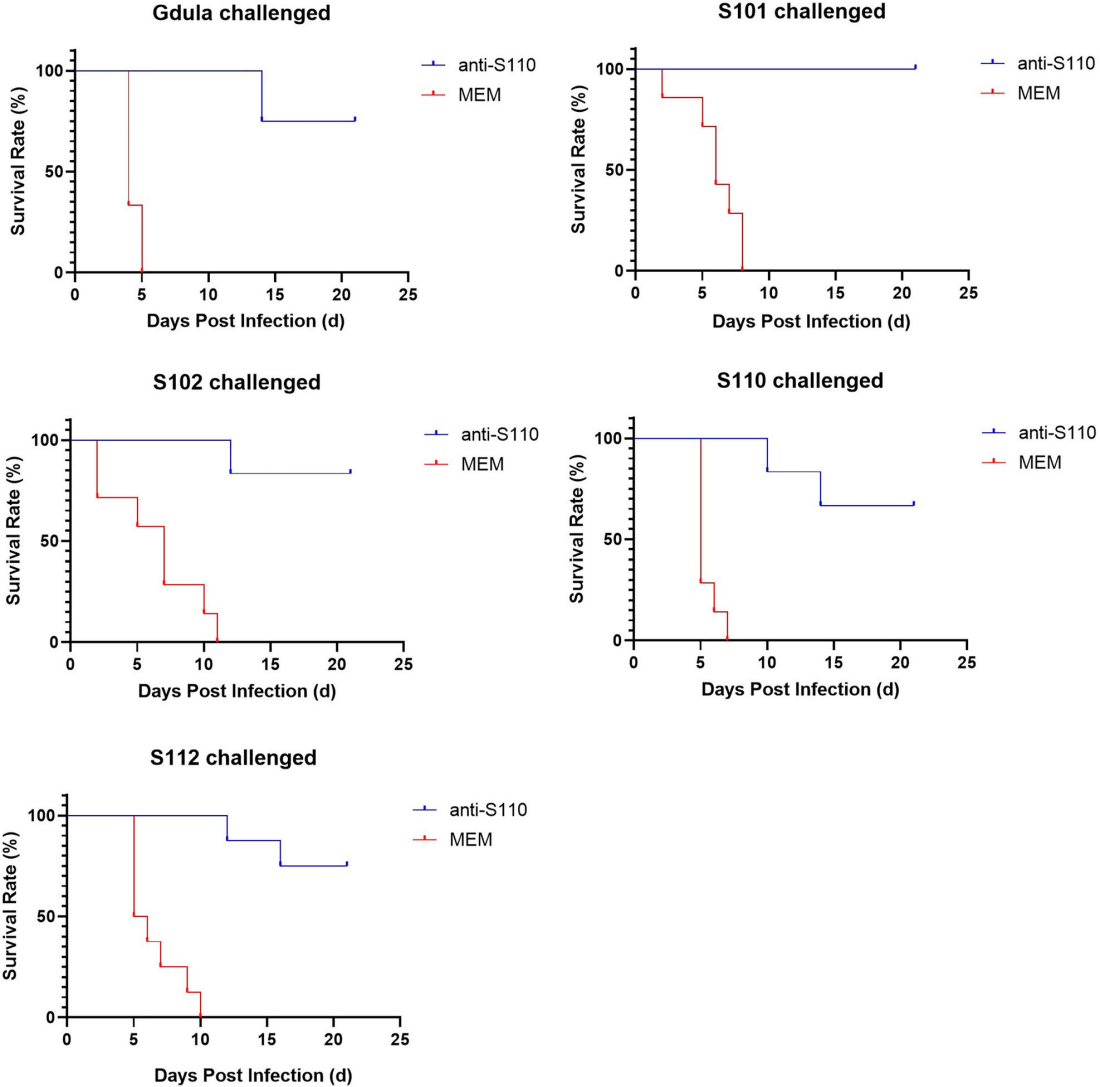
Cross protective effect of anti-S110 against lethal challenge of 5 CV-A6 strains. Anti-S110 (48-fold dilution) showed a good protective effect against 5 CV-A6 strains, and it ascended from 67% to 100% in the order S110, Gdula, S112, S102, and S101.

To verify the *in vivo* cross-neutralization ability of serum immunized with S110, the anti-S110 serum was diluted 48-fold. One-day-old BALB/c mice were intraperitoneally challenged with lethal doses (30 LD_50_) of Gdula, S101, S102, S110, or S112. A diluted anti-S110 serum was injected intraperitoneally within 1 h of the challenge. The results presented in Table 4 and Figure 5 demonstrate that a 48-fold dilution of anti-S110 exhibited a protective effect against lethal doses of all five CV-A6 strains (survival rates ranging from 67% to 100%), indicating its broad-spectrum cross-protective efficacy *in vivo*, which is consistent with the findings observed *in vitro*.

**Table 4.** Cross-protection of anti-S110 against the lethal challenge of five CV-A6 strains.

Item	CV-A6 strains				
	Gdula	S101	S110	S112	S102
NtAb titer *in vitro*	1536	3072	6144	2048	4096
Survival rate *in vivo* (%)	75	100	67	75	83

## Discussion

All the CV-A6 strains were divided into several subgenotypes. However, no uniform standards have yet been established for this classification. Identifying of distinct CV-A6 genotypes, categorized as A-D [58] or A-F [59], relies on analyzing the VP1 sequence, with a primary sequence difference exceeding 15% serving as an indicator. Genotype B was further classified as subgenotype B1 and B2, whereas genotype D was subdivided into subgenotypes D1–D3 [58]. The Gdula strain (GenBank ID: AY421764), identified as genotype A, was the first isolated CV-A6 strain in the USA in 1949 [58, 60]. However, it is not prevalent in the general population. The VP1 sequences of CV-A6 strains from multiple countries after 2008 revealed that, except for the Indian strain N-313 (subgenotype C2), all other international CV-A6 strains belonged to genotype D [58]. The outbreaks of CV-A6 observed in Europe from 2008 to 2010 can be attributed to two major subgenotypes (D1 and D3), with subgenotype D3 emerging as the predominant cause of most global outbreaks after 2010 [58]. In recent years, subgenotype D3 has caused HFMD outbreaks in Thailand [24], India [27], Brazil [3,19], and Spain [39,40]. In China, research [61] indicates that most CVA6 strains belong to the D genotype (95.6%), which mainly circulates in the eastern, northern, and southern regions of China. Subgenotype D3 first circulated in 2008 and has become the predominant subgenotype since 2009, reaching a peak in 2013, whereas D2 has been mostly undetectable in recent years [61]. Therefore, subgenotype D3 viruses are likely to exhibit enhanced transmissibility, infectivity, and virulence, potentially serving as the primary driver of sustained global circulation of CV-A6 [58]. To effectively implement outbreak prevention and control, various vaccine R&D institutions and manufacturers have selected subgenotype D3 (Table 2, Figure 2) as a candidate vaccine strain isolated from HFMD cases in three regions of mainland China (Guangdong Province, Hubei Province, and Beijing) from 2016 to 2019. Our results showed that the homology in the VP1 sequence of subgenotype D3 ranged from 94.21% to 99.78%. The homology between subgenotypes D3 and D1 isolated from Chinese Taiwan in 2007 was 88.52%–90.05%, and 82.19%–83.61% with prototype A/Gdula, suggesting that they are largely different from genotype A.

Because of differences in the use of detection strains, comparing the efficacy of CV-A6 vaccines produced by manufacturers in China and several Asia-Pacific countries and regions is challenging. Previous studies have demonstrated that the choice of the detection strain used in the neutralization assay can significantly affect the NtAb titer [51,56]. Although selecting an appropriate detection strain for estimating NtAb titers is crucial in vaccine R&D, it has not received adequate attention. To compare the differences in the test results caused by the different CV-A6 strains, we set the serum NtAb titer against the corresponding viral strain to 100. This approach helps minimize errors resulting from fluctuations in serum titers [51,56]. The MAX/MIN values of all strains against the sera were analyzed, assuming that a lower value indicated better homogeneity of detection. Our results showed that S112 exhibited the highest GMT (132.0) and lowest MAX/MIN value (48.0). Therefore, the S112 strain is more suitable as a detection strain for CV-A6 neutralization antibodies because it minimizes the bias introduced by other detection strains. This strain plays a crucial role in vaccine evaluation and provides a solid foundation for comparing various CV-A6 vaccines. Additionally, S112 exhibited the highest LD_50_ (4.23 lgCCID_50_/mL) in mice, making it a potential challenge strain for future animal protection models *in vivo*, which may be used to evaluate *in vivo* efficacy and correlate with *in vitro* neutralization potency.

A significant effect of each strain on the NtAb test (MAX/MIN_S112 _= 48.0, MAX/MIN_S103 _= 16410.3) was observed. The large difference in MAX/MIN values suggests that there may be differences in the neutralization epitopes of these variants. However, studies of the neutralizing epitope of CV-A6 are limited. The information provided in Supplementary Table 1 reveals the identification of two CV-A6-specific linear B-cell epitopes, which were mapped to the GH loop (206–220 aa) and the C-terminal region (291–305 aa) of VP [62]. Upon comparison of the VP1 sequences of the 15 CV-A6 strains, hardly any variation was observed in the aforementioned regions of VP1 (Supplementary Figure 1), indicating the presence of additional neutralizing epitopes in CV-A6. Another study indicated that the near-atomic-resolution structure of CV-A6 A-particle complexed with a neutralizing antibody mapped an immune-dominant neutralizing epitope (mAb-1D5) onto the surface loops (BC, EF, HI, and DE loops) of VP1 [63]. We compared the VP1 amino acid sequences of 15 CV-A6 strains with the 12 critical residues in the VP1 epitope of CV-A6 identified by mAb-1D5 [63] and observed a difference (D or N) at 138aa within the DE loop region (Supplementary Table 2). However, this change was not associated with cross-neutralization activity. The binding activities of S112 and S103 were evaluated using five mAbs (1D5 [63], 2B5, 4D6, 7F9, and 6C8) that target the conformational epitopes of CV-A6 with high neutralizing activity. A significant difference in the binding activity to mAb-2B5 was observed between S112 and S103 (*P* = 0.0066) (Supplementary Figure 2). We also compared the amino acid sequences of VP1, VP2, and VP3 between S112 and S103, which revealed a discrepancy of four amino acids (Supplementary Table 3). The differential sites in the capsid protein may serve as crucial determinants of the disparities in cross-neutralization activity. The epitope of 2B5 remains unclear, and further studies are needed to determine whether it is associated with these four amino acids. The interaction between CV-A6 and the receptor KRMEN1 remains unresolved, leaving uncertainty regarding whether variations in receptor-binding sites contribute to differences in cross-neutralization activity. Further research will be conducted on the structural characteristics of the CV-A6 particles complexed with mAb-2B5 and the receptor KRMEN1 to investigate whether this discovery can explain the variation in cross-neutralization activity among different strains of CV-A6.

In addition, the evaluation of cross-neutralization activity is a pivotal indicator of the broad-spectrum efficacy of vaccine strains [64,65]. During the initial stages of EV-A71 vaccine development, the NIFDC spearheaded cross-neutralization studies of diverse candidate vaccine strains obtained from relevant manufacturers, assisting manufacturers in selecting strains that exhibited exceptional cross-neutralization capabilities across different genotypes and within the same genotype [51,66]. Therefore, an effective EV71 vaccine was successfully developed [43,44,67]. In this study, we collaborated with Chinese CV-A6 vaccine manufacturers to provide *in vitro* and *in vivo* cross-neutralization data, which served as a reference for assessing the broad-spectrum efficacy of the vaccine strains. Fifteen CV-A6 strains were collected and cross-neutralization of CV-A6 strains was conducted to understand the differences in the cross-neutralization ability of different CV-A6 genotypes. The results showed that anti-S110 had good cross-neutralization activity against genotype A and subgenotypes D1 and D3, with the highest GMT (3153.3) and lowest MAX/MIN value (16.0). The anti-S110 serum was diluted 1:48 to investigate its protective effects against lethal challenge in mice. The results showed that anti-S110 serum had good protection against five lethal doses of viruses (including type A and D3 subtypes; the protection rate was 67%–100%), which proved that anti-S110 serum also had broad-spectrum cross-protection ability *in vivo*. Therefore, our study provides valuable reference data for manufacturers regarding the broad-spectrum efficacy of these strains.

Our study provides a framework for selecting detection strains to estimate NtAbs, which is crucial for comparing different vaccines. The cross-neutralization activity data in this study originated primarily from animal models, lacking human vaccination data. Therefore, as the CV-A6 vaccine progresses to clinical trials, we will assess the suitability of the detection strain for NtAb testing in humans.

## Supplementary Material

Supplementary_tables

Supplementary_figures
