# Supplementary material for: A screening study on the detection strain of Coxsackievirus A6: the key to evaluating neutralizing antibodies in vaccines
Source: Emerg Microbes Infect. 2024 Feb 23;13(1):2322671. doi: 10.1080/22221751.2024.2322671 (PMC10906128; doi:10.1080/22221751.2024.2322671)
Supplement: Supplementary_tables [file TEMI_A_2322671_SM9240.zip › Supplementary table 1.docx]

**Supplementary Table 1.** Linear and conformational epitopes of CV-A6

| VP1 domains | Peptide | Residues (aa) | Antibody | Ref. |
| --- | --- | --- | --- | --- |
| GH loop | P42 | 206–220 | Mouse antiserum | [62] |
| C-terminal | P59 | 291–305 | Mouse antiserum | [62] |
| BC, EF, HI, DE loops | / | BC loop: 96–99  EF loop: 159, 161,162,164  HI loop: 236,237,239  DE loop: 138 | mAb 1D5 | [63] |
