# Supplementary material for: A screening study on the detection strain of Coxsackievirus A6: the key to evaluating neutralizing antibodies in vaccines
Source: Emerg Microbes Infect. 2024 Feb 23;13(1):2322671. doi: 10.1080/22221751.2024.2322671 (PMC10906128; doi:10.1080/22221751.2024.2322671)
Supplement: Supplementary_tables [file TEMI_A_2322671_SM9240.zip › Supplementary table 2.docx]

Supplementary Table 2. mAb-1D5 targets the binding epitope of CV-A6 strains

| Name | Critical residues in the epitope of VP1 [63] | | | | | | | | | | | | | | |  |
| --- | --- | --- | --- | --- | --- | --- | --- | --- | --- | --- | --- | --- | --- | --- | --- | --- |
|  | BC loop | | | |  | EF loop | | | |  | HI loop | | |  | DE loop | |
|  | 96 | 97 | 98 | 99 |  | 159 | 161 | 162 | 164 |  | 236 | 237 | 239 |  | 138 | |
| Gdula | T | S | Q | D |  | D | R | K | Y |  | S | T | G |  | D | |
| XM | **·** | **·** | L | **·** |  | **·** | **·** | **·** | **·** |  | **·** | **·** | **·** |  | N | |
| S101 | **·** | **·** | L | **·** |  | **·** | **·** | **·** | **·** |  | **·** | **·** | **·** |  | N | |
| S102 | **·** | **·** | L | **·** |  | **·** | **·** | **·** | **·** |  | **·** | **·** | **·** |  | N | |
| S103 | **·** | **·** | L | **·** |  | **·** | **·** | **·** | **·** |  | **·** | **·** | **·** |  | N | |
| S104 | **·** | **·** | L | **·** |  | **·** | **·** | **·** | **·** |  | **·** | **·** | **·** |  | **·** | |
| S105 | **·** | **·** | L | **·** |  | **·** | **·** | **·** | **·** |  | **·** | **·** | **·** |  | **·** | |
| S106 | **·** | **·** | L | **·** |  | **·** | **·** | **·** | **·** |  | **·** | **·** | **·** |  | **·** | |
| S107 | **·** | **·** | L | **·** |  | **·** | **·** | **·** | **·** |  | **·** | **·** | **·** |  | N | |
| S108 | **·** | **·** | L | **·** |  | **·** | **·** | **·** | **·** |  | **·** | **·** | **·** |  | N | |
| S109 | **·** | **·** | L | **·** |  | **·** | **·** | **·** | **·** |  | **·** | **·** | **·** |  | **·** | |
| S110 | **·** | **·** | L | **·** |  | **·** | **·** | **·** | **·** |  | **·** | **·** | **·** |  | N | |
| S112 | **·** | **·** | L | **·** |  | **·** | **·** | **·** | **·** |  | **·** | **·** | **·** |  | N | |
| S113 | **·** | **·** | L | **·** |  | **·** | **·** | **·** | **·** |  | **·** | **·** | **·** |  | **·** | |
| S114 | **·** | **·** | L | **·** |  | **·** | **·** | **·** | **·** |  | **·** | **·** | **·** |  | N | |
| Ref.63 | **·** | **·** | L | **·** |  | **·** | **·** | **·** | **·** |  | **·** | **·** | **·** |  | N | |
