# Supplementary material for: A screening study on the detection strain of Coxsackievirus A6: the key to evaluating neutralizing antibodies in vaccines
Source: Emerg Microbes Infect. 2024 Feb 23;13(1):2322671. doi: 10.1080/22221751.2024.2322671 (PMC10906128; doi:10.1080/22221751.2024.2322671)
Supplement: Supplementary_tables [file TEMI_A_2322671_SM9240.zip › Supplementary table 3.docx]

**Supplementary Table 3.** Differential amino acid sites at S103 and S112 in the P1 region

| Name | VP1 |  | VP2 |  | VP3 | |
| --- | --- | --- | --- | --- | --- | --- |
|  | C-terminal |  | EF loop |  | GH loop | β-sheet |
|  | 275 aa |  | 139 aa |  | 180 aa | 220 aa |
| S103 | S |  | T |  | I | K |
| S112 | G |  | M |  | T | R |
