# Supplementary figures and images for: A screening study on the detection strain of Coxsackievirus A6: the key to evaluating neutralizing antibodies in vaccines
Source: Emerg Microbes Infect. 2024 Feb 23;13(1):2322671. doi: 10.1080/22221751.2024.2322671 (PMC10906128; doi:10.1080/22221751.2024.2322671)

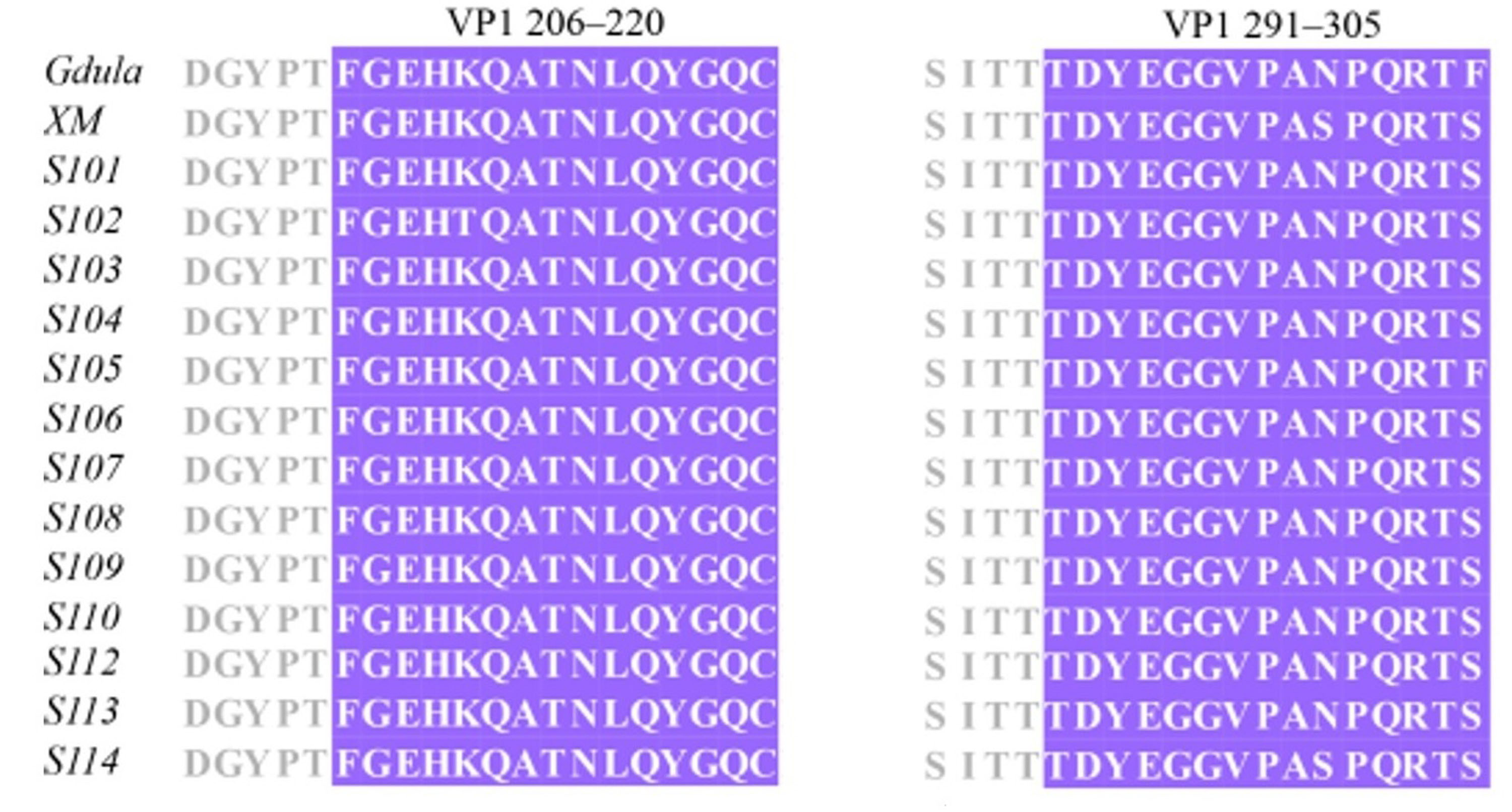

Supplement: Supplementary_figures [file TEMI_A_2322671_SM9237.zip › Supplementary fig 1.jpg]

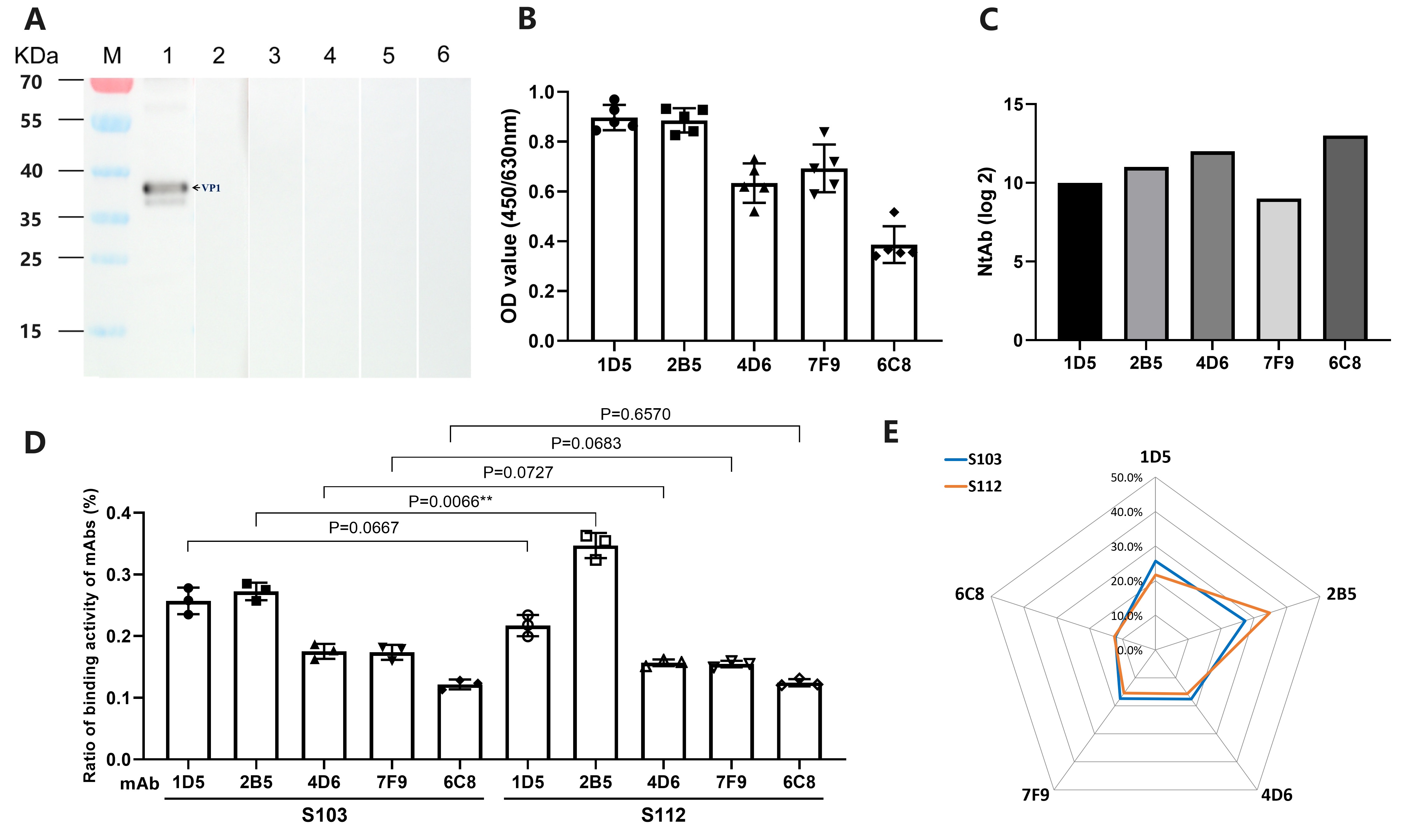

Supplement: Supplementary_figures [file TEMI_A_2322671_SM9237.zip › Supplementary fig 2.jpg]
